# Enhanced thermal effectiveness for electroosmosis modulated peristaltic flow of modified hybrid nanofluid with chemical reactions

**DOI:** 10.1038/s41598-022-17522-3

**Published:** 2022-08-12

**Authors:** Arafat Hussain, Jun Wang, Yasir Akbar, Riaz Shah

**Affiliations:** 1 Institute of Applied System Analysis, Jiangsu University, Zhenjiang, 212013 Jiangsu People’s Republic of China; 2 Department of Mathematics, COMSATS University Islamabad, Islamabad, Pakistan

**Keywords:** Biological physics, Fluid dynamics, Thermodynamics, Mechanical engineering, Nanoparticles, Magnetic properties and materials

## Abstract

In this analysis, the thermal and flow properties of modified hybrid nanofluids (MNFs) have been investigated under the effects of electroosmosis and homogeneous-heterogeneous chemical reactions. Three types of nanoparticles of *Cu*, *CuO*, and *Al*_*2*_*O*_*3*_ are utilized to monitor the performance of the MNFs with water as a working liquid. The determination of the heating phenomenon is explored by incorporating the effects of NPs shape, temperature reliant viscosity, Joule heating, heat generation/absorption and viscous dissipation. In this exploration, equal diffusion factors for the auto catalyst and reactants are assumed. The model formulation contains a highly non-linear PDE system, which is converted to ODEs under physical assumptions with lubrication and Debye–Huckel. The solution treatment involves the Homotopy perturbation method for solving the governing differential equations is used. A major outcome discloses that an addition in heterogeneous reaction parameter aids in enhancing the concentration profile. In a result, the temperature curve decreases at increasing volume fraction of the NPs. Modified hybrid NFs have higher heat transfer rate as compared to base *H*_*2*_*0*, or ordinary *Al*_*2*_*O*_*3*_*–H*_*2*_*0* and hybrid *Cu* + *Al*_*2*_*O*_*3*_*–H*_*2*_*0* NFs. Pressure gradient decreases by improving electroosmotic parameter. Further a comparison between analytically (HPM) and numerical results (NDSolve) show that both results are in good agreement.

## Introduction

Heat transport is considered one of the main and crucial phenomena in different areas of technologies. The thermal dynamics of nanofluids (NFs) is extremely exciting and new in terms of applications. NFs, an inevitable class of fluid with exceptional heat transfer capability due to suspended nanoscale particles in the base fluid. NFs alludes to a consistent mixture of minute metal particles (5–100 nm) with working liquids such as kerosene, water, oils, EG etc. The resulting liquids, called NFs, have excellent thermal conductivity^1–3^, uniformity, high stability, and low fouling, making them a universally used medium in various activities, embracing automotive, power generation, extrusion machinery, chemical production, solar collectors, air purifiers, electronics, nuclear systems. and drug therapy. Experimental studies also suggest that the TC of NFs depends on a range of aspects, such as volume fraction of particles, particle size, particle structure, base liquid material, clustering, additives, temperature, and acidity of the NFs^4,5^. In the nanoliquid size spectrum, the particle surface to particle volume ratio is so large that any interactions are driven by short-range forces such as surface forces and van der Waals attraction. Buongiorno^6^ investigated convective nanofluidic transportation while incorporating Brownian motion and thermophoresis into account. In his research, he noticed that Brownian and thermophoretic diffusion are key factors for extraordinary increase in heat transfer by the NFs. Tiwari and Das^7^ fashioned NFs transportation by entering the size of tiny materials, thermal conductivity, viscosity, and volume fraction into the NFs heat transfer mechanism. Lazarus^8^ addressed the applications of NFs in different heat transfer phenomena. Few recent studies conducted in this direction may also be observed through references^9–14^.

Oscillations due to transverse translational waves transmitting through a flexible wall led to uninterrupted periodic oscillations of muscle conductors, called peristalsis. This kind of flow is produced by a progressive wave moving from low pressure to high pressure along the tract boundaries under the action of a pump. Peristaltic flow is a phenomenon of natural transport in which body fluid moves from one location to another by continuous relaxation and muscle contraction. Peristaltic movement during the lubrication procedure has been extensively discussed by Shapiro et al.^15^. Akram et al.^16^ researched ramifications of nanoliquid on peristaltic transportation through an asymmetric channel. Abbasi et al.^17^ examined the second law analysis for peristaltic movement of H_2_O based nanoliquid. Analytical solution for the resulting system is obtained using HPM. Akbar et al.^18^ explored the implications of Hall current and radiative heat flux on peristaltic transportation of nanoliquid with irreversibility rate. Reddy et al.^19^ investigated the entropy rate for the gold-blood NFs flow in a microchannel.

Many chemical reactions occurring in various biological and physical phenomena occur in the presence of a catalyst^20^. The procedure is accelerated by using a catalyst without using it. Due to the physical state of substances, two chemical reactions occur, i.e., homogeneous, and heterogeneous chemical reactions. In addition, these reactions are classified as single phase (gas, liquid and solid) and are called homogeneous reactions, while heterogeneous reactions occur at two or more phases when one or more reactants undertake chemical modifications such as (liquid, solid, solid and gas,). Some reactions are unable to proceed on their own or are conducted with the participation of any catalyst. Several analyses are published on chemical reactions for different uses^21–23^.

Electroosmotic flow (EOF) relative to a fixed charged surface is the movement of an ionized fluid under the impact of an applied potential or an external electric field. This impact has continued to receive much attention in recent decades due to its application in micropumps, small scale liquid handling, and efficient design of mass and heat transfer systems. Some of the pioneering results in the field of microfluidics are the evolution of inkjet printheads, DNA chip sequencing, drug supply for cancer patients, lab-on-a-chip technologies, and microthermal technologies. Such applications, which include soil conditioning and microscale chemical separation, have encouraged many scientists to research electroosmotic flow in micro geometries over the years. A few representative debates on electroosmosis have been conducted in the investigations^24–34^.

Inspired by the previously mentioned inspirations, the present pursual aims to investigate the efficiency of heat transfer and flow of modified hybrid nanofluid under the effects of heterogeneous and homogeneous chemical reactions, temperature-dependent viscosity, electric and magnetic fields, and heat generation/absorption. In addition, shape aspects are also being studied for the nanomaterials used. The MNPs are composed of three forms of nanomaterials, i.e., *Cu*, *Al*_*2*_*O*_*3*_ and *CuO*, NPs which are used to evaluate thermal performance. Basic assumptions are used to design defining expressions for the flow model. The analytical computation using Homotopy Perturbation Method (HPM) is performed for solution procedure. A comprehensive analysis of the corresponding parameters on the flow properties, thermal aspects of nanomaterials and shape features are described and reflected through graphs and tables.

## Problem formulation

**Figure 1:**
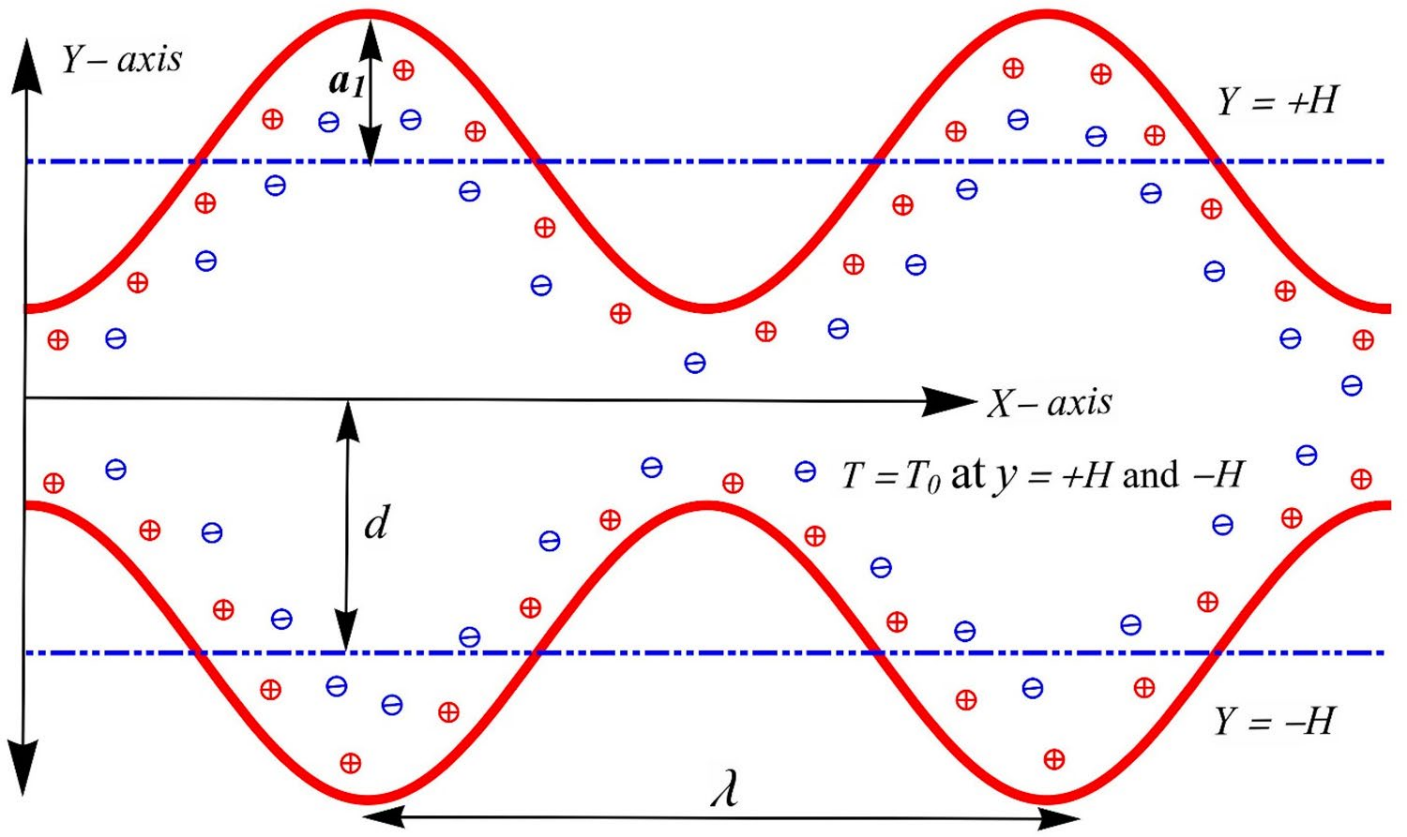
Geometry of the considered problem.

Here the flow of an electrically conductive modified hybrid nanofluid i.e., *Al*_*2*_*O*_*3*_, *CuO*, *Cu* nanomaterials, suspended in aqueous (water) ionic solution, which is propelled by the combined effects of electroosmosis and dissemination of sine waves along the entire length of the channel walls with a constant speed *c* is considered. We assumed that the walls of the channel are flexible, on which migrating waves of a sinusoidal nature, having a large wavelength, are superimposed. Cartesian coordinates (*x*, *y*) are utilized, the *y* and *x*-*axes* are set aside along the normal position and center line, respectively. An external electric field is applied across the EDL in the *x-axis* direction to generate electroosmotic forces. The modified hybrid nanofluid is prepared by mixing 1% volume fraction of solid nanoparticles of *Al*_*2*_*O*_*3*_, 1% volume proportion of *CuO* and 1% of *Cu* in an aqueous (water) solution. The analysis is conducted in the presence of variable viscosity, magnetic field, viscous dissipation, heterogeneous and homogeneous chemical reactions, and Joule heating. Mathematically, peristaltic walls are given as^18^:

1 $$\pm \overline{H}(\overline{X},\overline{t}) = \pm a_{1} \cos \left( {\frac{2\pi }{\lambda }(\overline{X} - c\overline{t})} \right) \pm d.$$

where $- \overline{H}(\overline{X},\overline{t})$ and $+ \overline{H}(\overline{X},\overline{t})$ allocates for the lower and upper walls respectively (see Fig. 1). Further, a simple template for the interaction between a heterogeneous (or surface) reaction and homogeneous (or bulk) reaction in which two chemical species *A* and *B* are presumed. A homogeneous reaction can be expressed by cubic autocatalysis, given by the^22^:

2 $$A + 2B \to 3B,{\text{ rate}} = k_{c} \alpha \beta^{2} ,$$

and that the heterogeneous reaction is^22^,

3 $$A \to B,{\text{ rate}} = k_{s} \alpha .$$

Here $\alpha$ and $\beta$ are the concentrations of the species $A$ and $B$, respectively. ${k}_{j}\left(j=s,c\right)$ are the constants rate. It is also supposed that these two reaction processes are single, first-order, isothermal in the catalyst. It is important to note here that these two reactions proceed at the same temperature.

### Electro and magnetohydrodynamics

Generalized Ohmic law is given as^25^:

4 $${\mathbf{J}} = \sigma_{mnf } [{\mathbf{E}} + {\mathbf{V}} \times {\mathbf{B}}].$$

In this problem ***E*** = [*E*_*x,*_* 0, 0*] and ***B*** = [*0, B*_*0*_*,0*]. Lorentz force using Eq. (4) is becoming:

5 $${\mathbf{J}} \times {\mathbf{B}} = \left[ { - A_{4} \overline{U}B_{0}^{2} \sigma_{f} ,A_{4} B_{0} \sigma_{f} E_{x} ,0} \right].$$

The Poisson’s equation^25^ for a symmetric channel is characterized as:

6 $$\nabla^{2} {\overline{\Omega }} = - \frac{{\rho_{e} }}{\varepsilon }.$$

The net charge density (${\rho }_{e}$) obeys Boltzmann distribution^25^ are defined as:

7 $$\rho_{e} = ez\left( {\overline{n}_{ + } - \overline{n}_{ - } } \right),$$

the cations and anions are specified as^33^:

8 $$\overline{n}_{ \pm } = \overline{n}_{0} {\text{e}}^{{\left( { \pm \frac{ez}{{T_{av} K_{B} }}{\overline{\Omega }}} \right)}} .$$

Using Equations. (7) and (8) in (6) and the implementation of the Debye-Hückel approximation^34^:

9 $$\frac{{d^{2} {\Omega }}}{{dz^{2} }} = \omega^{2} {\Omega ,}$$

with boundary conditions^33^:

10 $$\begin{array}{*{20}c} {{\Omega }(y) = 1,{\text{ at }}y = 0,} \\ {{\Omega }(y) = 0,{\text{ at }}y = h.} \\ \end{array}$$

where $\omega$ is an electroosmotic parameter. It is expressed as:

11 $$\omega = \frac{d}{{\lambda_{D} }},$$

where,

12 $$\lambda_{D} = \frac{1}{ez}\left( {\frac{{\varepsilon K_{B} T_{av} }}{{2n_{0} }}} \right)^{\frac{1}{2}} .$$

The analytical solution of Eq. (9) subject to boundary conditions (10) takes the resulting form:

13 $${\Omega }(y) = \frac{{\sinh \left( {\omega y} \right)}}{{\sinh \left( {\omega h} \right)}}.$$

The governing equations for the current flow configuration are written as^17,18,21,28^:

14 $$\frac{{\partial \overline{U}}}{{\partial \overline{X}}} + \frac{{\partial \overline{V}}}{{\partial \overline{Y}}} = 0,$$

15 $$\begin{gathered} \rho_{mnf} \left( {\overline{U}\frac{{\partial \overline{U}}}{{\partial \overline{X}}} + \frac{{\partial \overline{U}}}{{\partial \overline{t}}} + \overline{V}\frac{{\partial \overline{U}}}{{\partial \overline{Y}}}} \right) = 2\frac{\partial }{{\partial \overline{X}}}\left( {\mu_{mnf} \frac{{\partial \overline{U}}}{{\partial \overline{X}}}} \right) + \frac{\partial }{{\partial \overline{Y}}}\left( {\mu_{mnf} \left( {\frac{{\partial \overline{V}}}{{\partial \overline{X}}} + \frac{{\partial \overline{U}}}{{\partial \overline{Y}}}} \right)} \right) - \frac{{\partial \overline{P}}}{{\partial \overline{X}}} \hfill \\ \, - \rho_{e} \overline{E}_{x} + A_{4} \sigma_{f} \overline{U}B_{0} , \hfill \\ \end{gathered}$$

16 $$\rho_{mnf} \left( {\overline{U}\frac{{\partial \overline{V}}}{{\partial \overline{X}}} + \overline{V}\frac{{\partial \overline{V}}}{{\partial \overline{Y}}} + \frac{{\partial \overline{V}}}{{\partial \overline{t}}}} \right) = 2\frac{\partial }{{\partial \overline{Y}}}\left( {\mu_{mnf } \frac{{\partial \overline{V}}}{{\partial \overline{Y}}}} \right) - \frac{{\partial \overline{P}}}{{\partial \overline{Y}}} + \frac{\partial }{{\partial \overline{X}}}\left( {\mu_{mnf } \left( {\frac{{\partial \overline{V}}}{{\partial \overline{X}}} + \frac{{\partial \overline{U}}}{{\partial \overline{Y}}}} \right)} \right),$$

17 $$\begin{gathered} \left( {\rho C} \right)_{mnf} \left( {\frac{{\partial \overline{T}}}{{\partial \overline{t}}} + \overline{U}\frac{{\partial \overline{T}}}{{\partial \overline{X}}} + \overline{V}\frac{{\partial \overline{T}}}{{\partial \overline{Y}}}} \right) = K_{mnf} \nabla^{2} \overline{T} + \Phi + A_{4} \sigma_{f} \left( {\overline{E}_{x} } \right)^{2} + A_{4} \sigma_{f} \left( {\overline{U}B_{0} } \right)^{2} \hfill \\ \, \mu_{mnf} \left( {\left( {\frac{{\partial \overline{V}}}{{\partial \overline{X}}} + \frac{{\partial \overline{U}}}{{\partial \overline{Y}}}} \right)^{2} + 2\left( {\frac{{\partial \overline{U}}}{{\partial \overline{X}}}} \right)^{2} + 2\left( {\frac{{\partial \overline{V}}}{{\partial \overline{Y}}}} \right)^{2} } \right), \hfill \\ \end{gathered}$$

18 $$\frac{d\alpha }{{d\overline{t}}} = D_{A} \nabla^{2} \alpha - k_{c}^{2} \overline{\alpha }\beta^{2} ,$$

19 $$\frac{d\beta }{{d\overline{t}}} = D_{B} \nabla^{2} \beta + k_{c}^{2} \overline{\alpha }\beta^{2} .$$

In equations, $\rho_{mnf} , \, \overline{P}\left( {\overline{X},\overline{Y},\overline{t}} \right), \, \overline{T}, \, K_{mnf}$ and $\Phi$ represent the modified hybrid nanofluid density, pressure, temperature of nanoliquid, modified hybrid nanoliquid thermal conductivity and heat absorption, respectively (Table 1). For two-phase flows, the thermophysical properties i.e., density, heat capacity, dynamic viscosity, and electric conductivity of modified hybrid nanofluid are given as^14^:

20 $$\begin{gathered} \rho{_{mnf}} = \left( {1 - \phi_{3} } \right)\left\{ {\left( {1 - \phi_{2} } \right)\left[ {\left( {1 - \phi_{1} } \right)\rho_{f} + \phi_{1} \rho_{{p_{1} }} } \right] + \phi_{2} \rho_{{p_{2} }} } \right\} + \phi_{3} \rho_{{p_{3} }} , \hfill \\ \mu_{mnf} = \frac{{\mu_{f} }}{{\left( {1 - \phi_{1} } \right)^{2.5} \left( {1 - \phi_{2} } \right)^{2.5} \left( {1 - \phi_{3} } \right)^{2.5} }}{,} \hfill \\ \left( {\rho Cp} \right)_{mnf} = \left( {1 - \phi_{3} } \right)\left\{ {\left( {1 - \phi_{2} } \right)\left[ \begin{gathered} \left( {1 - \, \phi_{1} } \right)\left( {\rho C_{p} } \right)_{f} \hfill \\ + \phi_{1} \left( {\rho C_{p} } \right)_{{p_{1} }} \hfill \\ \end{gathered} \right] + \phi_{2} \left( {\rho C_{p} } \right)_{{p_{2} }} } \right\} + \phi_{3} \left( {\rho C_{p} } \right)_{{p_{3} }} , \hfill \\ \frac{{K_{mnf} }}{{K_{hnf} }} = \frac{{K_{{p_{3} }} + \left( {m - 1} \right)K_{hnf} - \left( {m - 1} \right)\phi_{3} \left( {k_{hnf} - k_{{p_{3} }} } \right)}}{{K_{{p_{3} }} + \left( {m - 1} \right)K_{hnf} + \phi_{3} \left( {k_{hnf} - k_{{p_{3} }} } \right)}}, \hfill \\ \end{gathered}$$

where

$$\frac{{k_{hnf} }}{{k_{nf} }} = \frac{{k_{{p_{2} }} + \left( {m - 1} \right)k_{nf} - \left( {m - 1} \right)\phi_{2} \left( {k_{nf} - k_{{p_{3} }} } \right)}}{{k_{{p_{2} }} + \left( {m - 1} \right)k_{nf} + \phi_{2} \left( {k_{nf} - k_{{p_{3} }} } \right)}}{,}$$

and

$$\frac{{k_{nf} }}{{k_{f} }} \, = \frac{{k_{{p_{1} }} + \left( {m - 1} \right)k_{f} - \left( {m - 1} \right)\phi_{1} \left( {k_{f} - k_{{p_{1} }} } \right)}}{{k_{{p_{3} }} + \left( {m - 1} \right)k_{f} + \phi_{1} \left( {k_{f} - k_{{p_{1} }} } \right)}},$$

$$\frac{{\sigma_{mnf} }}{{\sigma_{hnf} }} = \frac{{\sigma_{{p_{3} }} + 2\sigma_{hnf} - 2\phi_{3} \left( {\sigma_{hnf} - \sigma_{{p_{3} }} } \right)}}{{\sigma_{{p_{3} }} + 2\sigma_{hnf} + \phi_{3} \left( {\sigma_{hnf} - \sigma_{{p_{3} }} } \right)}}{, }$$

where

$$\frac{{\sigma_{hnf} }}{{\sigma_{nf} }} = \frac{{\sigma_{{p_{2} }} + 2\sigma_{nf} - 2\phi_{2} \left( {\sigma_{nf} - \sigma_{{p_{2} }} } \right)}}{{ \, \sigma_{{p_{2} }} + 2\sigma_{nf} + \phi_{2} \left( {\sigma_{nf} - \sigma_{{p_{2} }} } \right)}}, \,$$

and

$$\frac{{\sigma_{nf} }}{{\sigma_{f} }} = \frac{{\sigma_{{p_{1} }} + 2\sigma_{f} - 2\phi_{1} \left( {\sigma_{f} - \sigma_{{p_{1} }} } \right)}}{{\sigma_{{p_{1} }} + 2\sigma_{f} + \phi_{1} \left( {\sigma_{f} - \sigma_{{p_{1} }} } \right)}}.$$

**Table 1:**
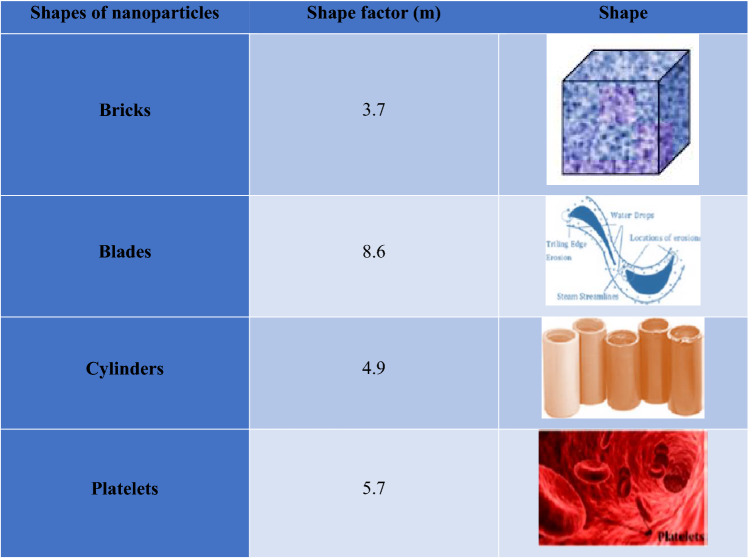
The shape factor for several types of NPs.

Numerical values of these properties are through Table 2. In Table 1, *ϕ*_1_, *ϕ*_2,_ *ϕ*_3_ is volume fractions of *CuO*, *Cu* and *Al*_2_*O*_3_ NPs. Subscripts *p*_1_*, p*_2_ and *p*_3_ denoting the *CuO, Cu* and *Al*_2_*O*_3_ nanoparticles. In addition, *m* is the shape factor for which numerical values are given in Table 1 for various shape factors.

**Table 2:** Numerical values of physical properties of base liquid and NPs.

Base fluid/solid particles	$\rho \left( {Kgm^{3} } \right)$	$C_{p} \left( {JKg^{ - 1} K^{ - 1} } \right)$	$k\left( {Wm^{ - 1} K^{ - 1} } \right)$	$\sigma \left( {\Omega m} \right)^{ - 1}$
$H_{2} O$	997.1	4179	0.613	0.05
$CuO\left( {\phi_{1} } \right)$	6500	540	18	$6.9 \times 10^{ - 2}$
$Cu\left( {\phi_{2} } \right) \,$	8933	385	400	$59.6 \times 10^{6}$
$Al_{2} O_{3} \left( {\phi_{3} } \right) \,$	3970	765	40	$35 \times 10^{6}$

The transformation between a fixed and movable frame of reference is listed as^18^:

21 $$\begin{gathered} \overline{x} = \overline{X} - c\overline{t} ,\overline{y} = \overline{Y},\beta = \overline{\beta },\alpha = \overline{\alpha },\overline{v}\left( {\overline{x},\overline{y}} \right) = \overline{V}\left( {\overline{X},\overline{Y},\overline{t}} \right), \hfill \\ \overline{u}\left( {\overline{x},\overline{y}} \right) = \overline{U}\left( {\overline{X},\overline{Y},\overline{t}} \right) - c, \, \overline{p}\left( {\overline{x},\overline{y}} \right) = \overline{P}\left( {\overline{X},\overline{Y},\overline{t}} \right). \hfill \\ \end{gathered}$$

Apply conversion to Eqs. (14)-(19), we get

22 $$\frac{{\partial \overline{u}}}{{\partial \overline{x}}} + \frac{{\partial \overline{v}}}{{\partial \overline{y}}} = 0,$$

23 $$\begin{gathered} \rho_{mnf} \left( {\left( {\overline{u} + c} \right)\frac{{\partial \overline{u}}}{{\partial \overline{x}}} + \overline{v}\frac{{\partial \overline{u}}}{{\partial \overline{y}}}} \right) = - \frac{{\partial \overline{p}}}{{\partial \overline{x}}} + \frac{\partial }{{\partial \overline{y}}}\left( {\mu_{mnf } \frac{{\partial \overline{v}}}{{\partial \overline{y}}}} \right) + 2\frac{\partial }{{\partial \overline{x}}}\left( {\mu_{mnf } \left( {\frac{{\partial \overline{v}}}{{\partial \overline{x}}} + \frac{{\partial \overline{u}}}{{\partial \overline{y}}}} \right)} \right) \hfill \\ \, - \rho_{e} \overline{E}_{x} + A_{4} \sigma_{f} \left( {\overline{u} + c} \right)B_{0} , \hfill \\ \end{gathered}$$

24 $$\rho_{hnf} \left( {\left( {\overline{u} + c} \right)\frac{{\partial \overline{v}}}{{\partial \overline{x}}} + \overline{v}\frac{{\partial \overline{v}}}{{\partial \overline{y}}}} \right) = - \frac{{\partial \overline{p}}}{{\partial \overline{y}}} + 2\frac{\partial }{{\partial \overline{y}}}\left( {\mu_{hnf } \frac{{\partial \overline{v}}}{{\partial \overline{y}}}} \right) + \frac{\partial }{{\partial \overline{x}}}\left( {\mu_{hnf } \left( {\frac{{\partial \overline{v}}}{{\partial \overline{x}}} + \frac{{\partial \overline{u}}}{{\partial \overline{y}}}} \right)} \right),$$

25 $$\begin{gathered} \left( {\rho C} \right)_{mnf} \left( {\left( {\overline{u} + c} \right)\frac{{\partial \overline{T}}}{{\partial \overline{x}}} + \overline{v}\frac{{\partial \overline{T}}}{{\partial \overline{y}}}} \right) = K_{mnf} \left( {\frac{{\partial^{2} \overline{T}}}{{\partial \overline{x}^{2} }} + \frac{{\partial^{2} \overline{T}}}{{\partial \overline{y}^{2} }}} \right) + A_{4} \sigma_{f} \left( {\overline{E}_{x} } \right)^{2} + A_{4} \sigma_{f} B_{0}^{2} \left( {\overline{u} + c} \right)^{2} \hfill \\ \, + \Phi + \mu_{mnf} \left( {\left( {\frac{{\partial \overline{v}}}{{\partial \overline{x}}} + \frac{{\partial \overline{u}}}{{\partial \overline{y}}}} \right)^{2} + 2\left( {\frac{{\partial \overline{u}}}{{\partial \overline{x}}}} \right)^{2} + 2\left( {\frac{{\partial \overline{v}}}{{\partial \overline{y}}}} \right)^{2} } \right), \hfill \\ \end{gathered}$$

26 $$\left( {\overline{u} + c} \right)\frac{{\partial \overline{\alpha }}}{{\partial \overline{x}}} + \overline{v}\frac{{\partial \overline{\alpha }}}{{\partial \overline{y}}} = D_{A} \left( {\frac{{\partial^{2} \overline{\alpha }}}{{\partial \overline{x}^{2} }} + \frac{{\partial^{2} \overline{\alpha }}}{{\partial \overline{y}^{2} }}} \right) - k_{c}^{2} \overline{\alpha }\overline{\beta }^{2} ,$$

27 $$\left( {\overline{u} + c} \right)\frac{{\partial \overline{\beta }}}{{\partial \overline{x}}} + \overline{v}\frac{{\partial \overline{\beta }}}{{\partial \overline{y}}} = D_{B} \left( {\frac{{\partial^{2} \overline{\beta }}}{{\partial \overline{x}^{2} }} + \frac{{\partial^{2} \overline{\beta }}}{{\partial \overline{y}^{2} }}} \right) + k_{c}^{2} \overline{\alpha }\overline{\beta }^{2} ,$$

### Reynold’s viscosity model

The Reynolds viscosity model is defined as:

28 $$\mu_{mnf} \left( T \right) = \frac{{\mu_{0} \left( {1 - \alpha_{1} \left( {T - T_{0} } \right)} \right)}}{{\left( {1 - \phi_{1} } \right)^{2.5} \left( {1 - \phi_{2} } \right)^{2.5} \left( {1 - \phi_{3} } \right)^{2.5} }}.$$

Using the subsequent dimensionless quantities:

29 $$\begin{aligned} x & = \frac{{\overline{x}}}{\lambda }, \, y = \frac{{\overline{y}}}{d}, \, \theta = \frac{{T - T_{0} }}{{T_{0} }}, \, u = \frac{{\overline{u}}}{c}, \, Ec = \frac{{c^{2} }}{{C_{f} T_{0} }}, \, v = \frac{{\overline{v}}}{c\delta }, \, h = \frac{{\overline{H}}}{d}, \, a = \frac{{a{}_{1}}}{d}, \, p = \frac{{d^{2} \overline{p}}}{{c\lambda \mu_{0} }}, \\ \Pr & = \frac{{\mu_{0} C_{f} }}{{K_{f} }}, \, Br = \Pr E, \, \varepsilon = \frac{{d^{2} \Phi }}{{T_{0} k_{f} }}, \, M = \sqrt {\frac{{\sigma_{f} }}{{\mu_{0} }}} B_{0} d, \, Gr = \frac{{\rho_{f} \beta_{f} gT_{0} d^{2} }}{{\mu_{0} c}}, \, u = \frac{\partial \psi }{{\partial y}}, \\ U_{hs} & = - \frac{{\varepsilon \overline{E}_{x} }}{{\mu_{0} }}, \, S = \frac{{d^{2} \sigma_{f} \overline{E}_{x}^{2} }}{{k_{f} T_{0} }}, \, \overline{\alpha } = \frac{f}{{\alpha {}_{0}}}, \, \overline{\beta } = \frac{g}{{\alpha_{0} }}, \, K = \frac{{k_{c} d^{2} \alpha_{0}^{2} }}{\upsilon }, \, K_{s} = \frac{{k_{c} d}}{{D_{A} }}, \, Sc = \frac{\upsilon }{{D_{B} }}, \\ \upsilon & = \frac{{\mu_{0} }}{{\rho_{0} }}, \, \phi = \frac{{C - C_{0} }}{{C_{0} }}, \, \xi = \frac{{D_{B} }}{{D_{A} }}, \, v = - \frac{\partial \psi }{{\partial x}}. \\ \end{aligned}$$

Utilizing “long wavelength and low Reynolds number approximations”, Eqs. (22)-(27) have following form:

30 $$\frac{\partial p}{{\partial x}} = A_{1} \frac{\partial }{\partial y}\left[ {\left( {1 - \alpha \theta } \right)\frac{{\partial^{2} \psi }}{{\partial y^{2} }}} \right] + U_{hs} \Omega^{\prime\prime}\left( y \right) - A_{4} M^{2} \left( {\frac{\partial \psi }{{\partial y}} + 1} \right) = 0,$$

31 $$\frac{\partial p}{{\partial y}} = 0,$$

32 $$A_{3} \frac{{\partial^{2} \theta }}{{\partial y^{2} }} + A_{1} Br\left( {1 - \alpha \theta } \right)\left( {\frac{{\partial^{2} \psi }}{{\partial y^{2} }}} \right)^{2} + A_{4} BrM^{2} \left( {\frac{\partial \psi }{{\partial y}} + 1} \right)^{2} + A_{4} S + \varepsilon = 0,$$

33 $$\frac{1}{Sc}\frac{{\partial^{2} f}}{{\partial y^{2} }} - Kfg^{2} = 0,$$

34 $$\frac{\xi }{Sc}\frac{{\partial^{2} g}}{{\partial y^{2} }} + Kfg^{2} = 0.$$

$A_{1}$, $A_{3}$, and $A_{4}$ are defined as:

$$A_{1} = \frac{1}{{\left( {1 - \phi_{1} } \right)^{2.5} \left( {1 - \phi_{2} } \right)^{2.5} \left( {1 - \phi_{3} } \right)^{2.5} }}{, }$$

35 $$\begin{gathered} A_{3} = \frac{{k_{mnf} }}{{k_{f} }} = \frac{{k_{{s_{3} }} + (m - 1)k_{hnf} - (m - 1)\phi_{3} \left( {k_{hnf} - k_{{s_{3} }} } \right)}}{{k_{{s_{3} }} + (m - 1)k_{hnf} + \phi_{3} \left( {k_{hnf} - k_{{s_{3} }} } \right)}} \times \frac{{k_{{s_{2} }} + (m - 1)k_{nf} - (m - 1)\phi_{2} \left( {k_{nf} - k_{{s_{3} }} } \right)}}{{k_{s2} + (m - 1)k_{nf} + \phi_{2} \left( {k_{nf} - k_{{s_{3} }} } \right)}} \hfill \\ \, \times \frac{{k_{{s_{2} }} + (m - 1)k_{nf} - (m - 1)\phi_{2} \left( {k_{nf} - k_{{s_{3} }} } \right)}}{{k_{{s_{2} }} + (m - 1)k_{nf} + \phi_{2} \left( {k_{nf} - k_{{s_{3} }} } \right)}}, \hfill \\ \end{gathered}$$

and

$$\begin{gathered} A_{4} = \frac{{\sigma_{mnf} }}{{\sigma_{f} }} = \frac{{\sigma_{{s_{3} }} + 2\sigma_{hnf} - 2\phi_{3} \left( {\sigma_{hnf} - \sigma_{{s_{3} }} } \right)}}{{\sigma_{{s_{3} }} + 2\sigma_{hnf} + \phi_{3} \left( {\sigma_{hnf} - \sigma_{{s_{3} }} } \right)}} \times \frac{{\sigma_{{s_{2} }} + 2\sigma_{nf} - 2\phi_{2} \left( {\sigma_{nf} - \sigma_{{s_{2} }} } \right)}}{{\sigma_{{s_{2} }} + 2\sigma_{nf} + \phi_{2} \left( {\sigma_{nf} - \sigma_{{s_{2} }} } \right)}} \hfill \\ \, \times \frac{{\sigma_{{s_{1} }} - 2\phi_{1} \left( {\sigma_{f} - \sigma_{{s_{1} }} } \right) + 2\sigma_{f} }}{{\sigma_{{s_{1} }} + \phi_{1} \left( {\sigma_{f} - \sigma_{{s_{1} }} } \right) + 2\sigma_{f} }}. \hfill \\ \end{gathered}$$

The dimensionless boundary conditions are listed as^34^:

36 $$\begin{gathered} \psi = 0, \, \frac{{\partial^{2} \psi }}{{\partial^{2} y}} = 0, \, \frac{\partial \theta }{{\partial y}} = 0,{\text{ at }}y = 0, \hfill \\ \psi = F, \, \frac{\partial \psi }{{\partial y}} = - 1, \, \theta = 0,{\text{ at }}y = h. \hfill \\ \, f{\text{ = 1, g}} = 0,{\text{ at }}y = 0, \hfill \\ \frac{\partial f}{{\partial y}} - K_{s} f = 0, \, \xi \frac{\partial g}{{\partial y}} + K_{s} g = 0,{\text{ at }}y = h. \hfill \\ \end{gathered}$$

The diffusion coefficients of chemical compounds *B* and *A* are not same in general. We can consider them equal in size as a particular case, and thus *D*_*A*_ = *D*_*B*_. Then Eqs. (33) and (34) result in the following relationship^21^:

37 $$f+g=1.$$

Hence

38 $$\frac{1}{Sc}\frac{{\partial }^{2}f}{\partial {y}^{2}}-Kf(1-f{)}^{2}=0,$$

and relevant boundary conditions turn into

39 $$f=1, \mathrm{ at } y=0,$$

$$\frac{\partial f}{\partial y}={K}_{s}f,at y=h.$$

Moreover, upon removal of pressure among Eqs. (30) and (31):

40 $$0 = A_{1} \frac{{\partial^{2} }}{{\partial y^{2} }}\left[ {\left( {1 - \alpha \theta } \right)\frac{{\partial^{2} \psi }}{{\partial y^{2} }}} \right] + U_{hs} \Omega^{\prime\prime\prime}\left( y \right) - A_{4} M^{2} \frac{{\partial^{2} \psi }}{{\partial y^{2} }} = 0.$$

### Solution methodology

The deemed Homotopy equation for the differential system has the following form:

41 $$H\left( {\psi ,p} \right) = \left( {1 - p} \right)\left[ {L_{1} \left( \psi \right) - L_{1} \left( {\psi_{0} } \right)} \right] + p\left[ \begin{gathered} A_{1} \frac{{\partial^{2} }}{{\partial y^{2} }}\left\{ {\left( {1 - \alpha \theta } \right)\frac{{\partial^{2} \psi }}{{\partial y^{2} }}} \right\} + \hfill \\ U_{hs} \Omega^{\prime\prime\prime}\left( y \right) - A_{4} M^{2} \frac{{\partial^{2} \psi }}{{\partial y^{2} }} \hfill \\ \end{gathered} \right],$$

42 $$H\left( {\theta ,p} \right) = \left( {1 - p} \right)\left[ {L_{2} \left( \theta \right) - L_{2} \left( {\theta_{0} } \right)} \right] + p\left[ \begin{gathered} A_{3} \frac{{\partial^{2} \theta }}{{\partial y^{2} }} + A_{1} Br\left( {1 - \alpha \theta } \right)\left( {\frac{{\partial^{2} \psi }}{{\partial y^{2} }}} \right)^{2} \hfill \\ + A_{4} BrM^{2} \left( {\frac{\partial \psi }{{\partial y}} + 1} \right)^{2} + A_{4} S + \varepsilon \hfill \\ \end{gathered} \right],$$

43 $$H\left( {\phi ,p} \right) = \left( {1 - p} \right)\left[ {L_{3} \left( \phi \right) - L_{3} \left( {\phi_{0} } \right)} \right] + p\left[ {\frac{1}{Sc}\frac{{\partial^{2} f}}{{\partial y^{2} }} - Kf\left( {1 - f} \right)^{2} } \right].$$

Relevant linear operators are received as:

44 $$L_{1} = A_{1} \frac{{\partial^{4} }}{{\partial y^{4} }} ,$$

45 $$L_{2} = A_{3} \frac{{\partial^{2} }}{{\partial y^{2} }} ,$$

46 $$L_{3} = \frac{{\partial^{2} }}{{\partial y^{2} }}.$$

Initial guesses are described as:

47 $$\psi_{0} = \frac{{3Fh^{2} y + h^{3} y - Fy^{3} - hy^{3} }}{{2h^{3} }},$$

48 $$\theta_{0} = 0,$$

49 $$f_{0} = \frac{1 - hL + Ly}{{1 - hL}}.$$

The series extension is expressed as:

50 $$\begin{gathered} \psi \left( {y,p} \right) = \psi_{0} + p\psi_{1} + p^{2} \psi_{2} + \cdots , \hfill \\ \theta \left( {y,p} \right) = \theta_{0} + p\theta_{1} + p^{2} \theta_{2} + \cdots , \hfill \\ f\left( {y,p} \right) = f_{0} + pf_{1} + p^{2} f_{2} + \cdots . \hfill \\ \end{gathered}$$

**Figure 2:**
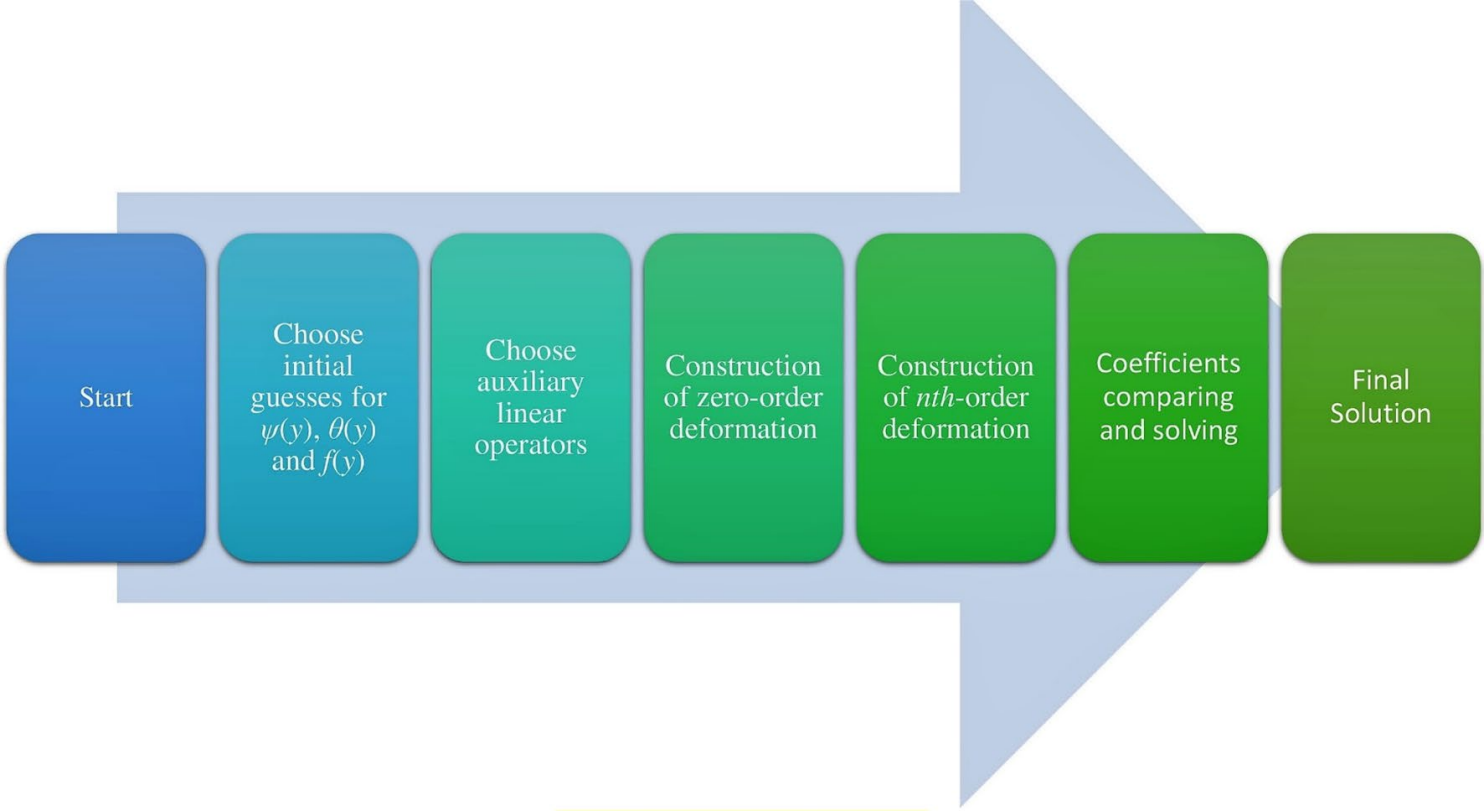
Flow chart of HPM.

The second, first and zeroth order systems of differential equations are achieved and then resolved with the assistance of Mathematica software. The HPM process is presented as a step-by-step diagram in Fig. 2.

### Zeroth order system

$$A_{1} \psi_{0}^{\left( 4 \right)} \left[ y \right] = 0,$$

$$\begin{gathered} A_{3} \theta_{0}^{\prime \prime } \left[ y \right] = 0, \hfill \\ f_{0}^{\prime \prime } \left[ y \right] = 0. \hfill \\ \end{gathered}$$

### First order system

$$\begin{gathered} US\omega^{3} \cosh \left[ {y\omega } \right]{\text{Csch}} \left[ {h\omega } \right] - A_{4} M^{2} \psi_{0}^{\prime \prime } \left[ y \right] - A_{1} \alpha \theta_{0}^{\prime \prime } \left[ y \right]\psi_{0}^{\prime \prime } \left[ y \right] - 2A_{1} \alpha \theta_{0}^{\prime } \left[ y \right]\psi_{0}^{\left( 3 \right)} \left[ y \right] - \hfill \\ \, A_{1} \alpha \theta_{0} \left[ y \right]\psi_{0}^{\left( 4 \right)} \left[ y \right] + A_{1} \psi_{1}^{\left( 4 \right)} \left[ y \right] = 0, \hfill \\ \end{gathered}$$

$$A_{4} S + \varepsilon + A_{4} BrM^{2} \left( {1 + \psi_{0}^{\prime } \left[ y \right]} \right)^{2} + A_{3} \theta_{1}^{\prime \prime } \left[ y \right] + A_{1} Br\left( {1 - \alpha \theta_{0} \left[ y \right]} \right)\psi_{0}^{\prime \prime } \left[ y \right]^{2} = 0,$$

$$- Kf_{0} \left[ y \right] + 2Kf_{0} \left[ y \right]^{2} - Kf_{0} \left[ y \right]^{3} - f_{0}^{\prime \prime } \left[ y \right] + \frac{{f_{0}^{\prime \prime } \left[ y \right]}}{Sc} + f_{1}^{\prime \prime } \left[ y \right] = 0.$$

Similarly, the second order system is achieved. Solutions for the above systems are analyzed using graphs and tables in the next section.

## Results and discussion

In this part, the impact of several relevant parameters in flow and heat transfer performance of a modified hybrid nanofluid flow through a symmetrical channel is analytically analyzed in detail.

### Concentration profile

**Figure 3:**
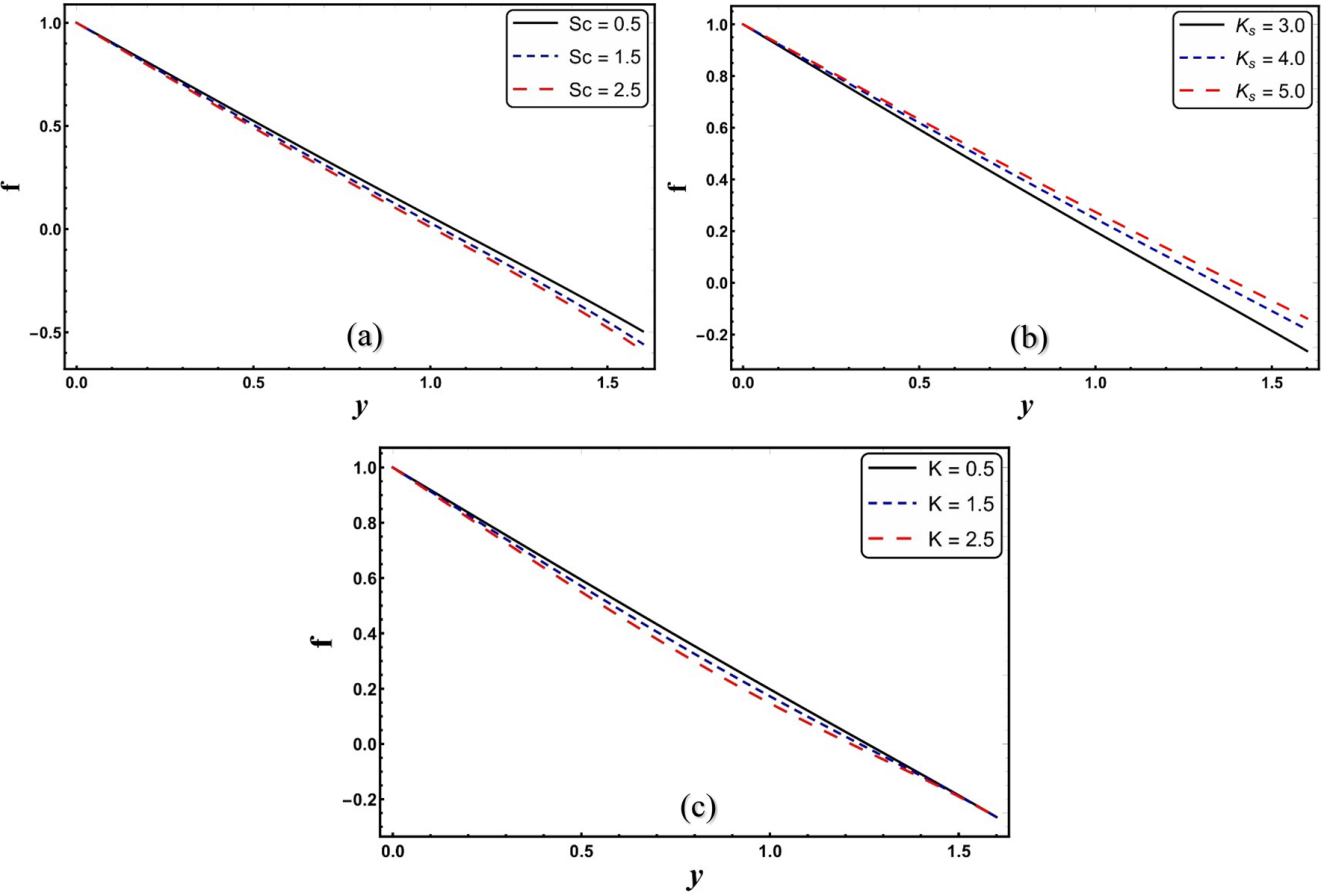
(**a**–**c**) Velocity profile for change in different embedded parameters.

Figures 3a–c display the results of concentration profile for change in *Sc*, *K*_*s,*_ and *K*. Figure 3a reveals that concentration profile decreases for higher *Sc*. Since the Schmidt number characterizes the flow of a liquid in which various processes of mass diffusion and momentum diffusion occur. This trend is in accordance with the Alarabi et al.^22^. Thus, higher *Sc* values reduce the rate of mass diffusion, which leads to the particles to scatter and thus a decrease in concentration is seen. It is observed from Fig. 3b that concentration enhances for larger values of heterogeneous reaction parameter. On the other hand, with a change in *K*, the reverse behavior in concentration is observed, as shown in Fig. 3c.

### Temperature profile

**Figure 4:**
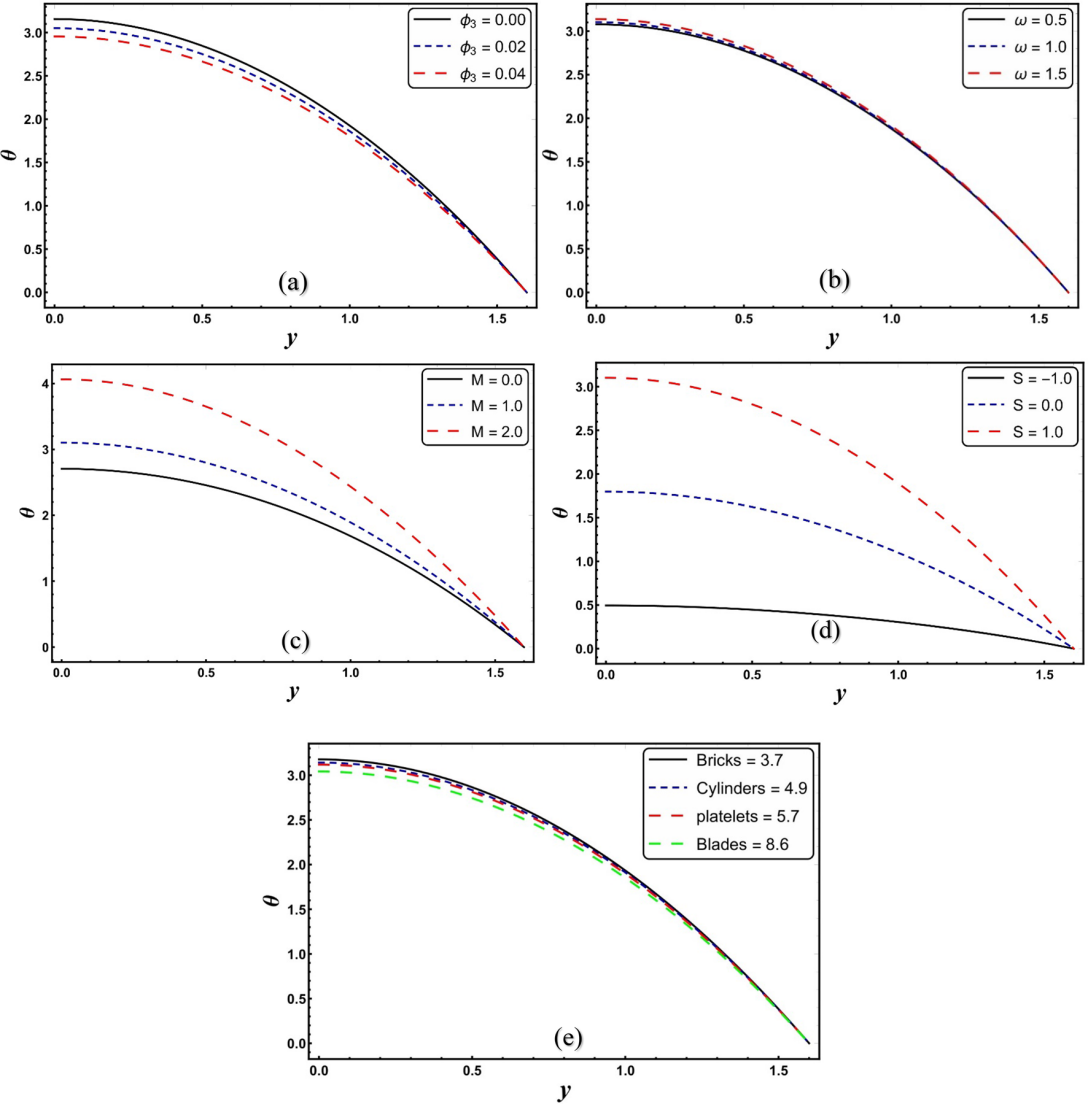
(**a**–**e**) *θ* for change in various parameters.

This subsection investigates the temperature of nanofluid containing modified hybrid nanoparticles (see Figs. 4a–e). Figure 4a predicts that the temperature curve of modified nanofluid decreases for a higher *ϕ*_*3*_. The addition of nanomaterials to the working liquid increases the heat transmission capability of the material. This leads to a reduction in temperature. Thus, the modified model is of immense significance to the mechanism of mechanical devices in which cooling agents are utilized. This finding is compatible with the Abbasi et al.^14^. Figure 4b demonstrates that temperature of modified nanofluid significantly elevates with incremental parameter *ω*. It is well understood that the electroosmotic force is a flow-resisting force that increases collisions between liquid particles. The internal kinetic energy of moving particles in a direction of flow increases as the frequency of collisions increases, resulting in an increase in temperature. Figure 4c shows that increasing S causes a significant increase in temperature of the modified nanofluid. Physically, this is due to the conversion of dissipated electrical energy into thermal energy. Similar trend is also encountered for higher *M* (see Fig. 4d). The geometry impact phenomena of nanomaterials are exemplified for various shapes, and this is reflected that varying the values of *m* give impact to various forms of NPs in the temperature field (see Fig. 4e). It is seen that brick-shaped NPs produce more heat than other NPs shapes. Brick-shaped NPs predominate compared to cylinder and plate-shaped NPs, while blade-shaped NPs give the minimum temperature.

### Heat transfer rate at the wall

Tables 3 is made to see the behavior of heat transfer rate at the wall $\left( { - \frac{{k_{mnf} }}{{k_{f} }}\theta^{\prime}\left( h \right)} \right)$ for various values of the governing parameters. Table 3 first column illustrates increased $- \frac{{k_{mnf} }}{{k_{f} }}\theta^{\prime}\left( h \right)$ with growth in nanoparticles volume fraction that is consistent with Akbar et al.^18^. The second column of Table 3 indicates that *ω* upsurges the $- \frac{{k_{mnf} }}{{k_{f} }}\theta^{\prime}\left( h \right)$ when it is installed in such a way that peristaltic pumping is assisted. Analogous behavior is noted for *S* (see third column). The fourth column shows that the $- \frac{{k_{mnf} }}{{k_{f} }}\theta^{\prime}\left( h \right)$ is increased by improving *M*. When a magnetic field is applied, the temperature of the modified hybrid nanofluid increases, thereby improving the heat transfer phenomenon at the wall. $- \frac{{k_{mnf} }}{{k_{f} }}\theta^{\prime}\left( h \right)$ is greater in blade-shaped NPs compared to other shaped NPs. Blade-shaped NPs are used to maintain heat transfer in technical systems.

**Table 3:** Numerical values of $\left( { - \frac{{k_{mnf} }}{{k_{f} }}\theta^{\prime}\left( h \right)} \right)$ against different involved parameters.

Parameters	Different shapes of nanoparticles						
$\phi_{3}$	$\omega$	$S$	$M$	Bricks	Cylinders	Platelets	Blades
0.01	1.0	1.0	1.0	4.0852	4.0853	4.0854	4.0858
0.02				4.1581	4.1584	4.1586	4.1593
0.04				4.3090	4.3096	4.3100	4.3111
	0.5			4.0797	4.0799	4.0800	4.0804
	1.0			4.0852	4.0853	4.0854	4.0858
	1.5			40,929	40,930	40,932	40,935
		−1.0		0.7008	0.7009	0.7009	0.7010
		0.0		2.3930	2.3931	2.3932	2.3934
		1.0		4.0852	4.0853	4.0854	4.0858
			0.0	3.6855	3.6857	3.6858	3.6861
			1.0	4.0852	4.0853	4.0854	4.0858
			2.0	5.1678	5.1680	5.1681	5.1686

### Comparison of heat transfer rate

Table 4 highlights the impact of base fluid (*H*_*2*_*0*), ordinary nanofluid (*Al*_2_*O*_3_-*H*_*2*_*0*), hybrid nanofluid (*Cu* + *Al*_2_*O*_3_-*H*_*2*_*0*) and modified hybrid nanofluid (*CuO* + *Cu* + *Al*_2_*O*_3_-*H*_*2*_*0*) on heat transfer rate. It is concluded that water with modified hybrid nanoparticles has higher heat transfer rate as compared to base fluid (*H*_*2*_*0*), ordinary nanofluid (*Al*_2_*O*_3_-*H*_*2*_*0*), hybrid nanofluid (*Cu* + *Al*_2_*O*_3_-*H*_*2*_*0*). This is due to increased thermal conductivity of the modified hybrid nanofluids.

**Table 4:** Comparison of $\left( { - \frac{{k_{mnf} }}{{k_{f} }}\theta^{\prime}\left( h \right)} \right)$ for regular base fluid, ordinary nanofluid, hybrid nanoliquid and modified hybrid nanoliquid.

*M*	Base fluid (*H*_*2*_*0*) *(ϕ*_1_ = *ϕ*_2_ = *ϕ*_3_ = 0)	Nanofluid (*Al*_2_*O*_3_-*H*_*2*_*0*) *(ϕ*_1_ = 0, *ϕ*_2_ = 0, *ϕ*_3_ = 0.03)	Hybrid nanofluid (*Cu* + *Al*_2_*O*_3_-*H*_*2*_*0*) *(ϕ*_1_ = 0.015, *ϕ*_3_ = 0.015, *ϕ*_2_ = 0)	Modified hybrid nanofluid (*CuO* + *Cu* + *Al*_2_*O*_3_-*H*_*2*_*0*) *(ϕ*_1_ = 0.01, *ϕ*_1_ = 0.01, *ϕ*_3_ = 0.01)
0.0	3.55898	3.58731	3.66059	3.68559
1.0	3.93591	3.96423	4.05441	4.08521
2.0	4.95542	4.99189	5.12308	5.1678

### Isotherms

**Figure 5:**
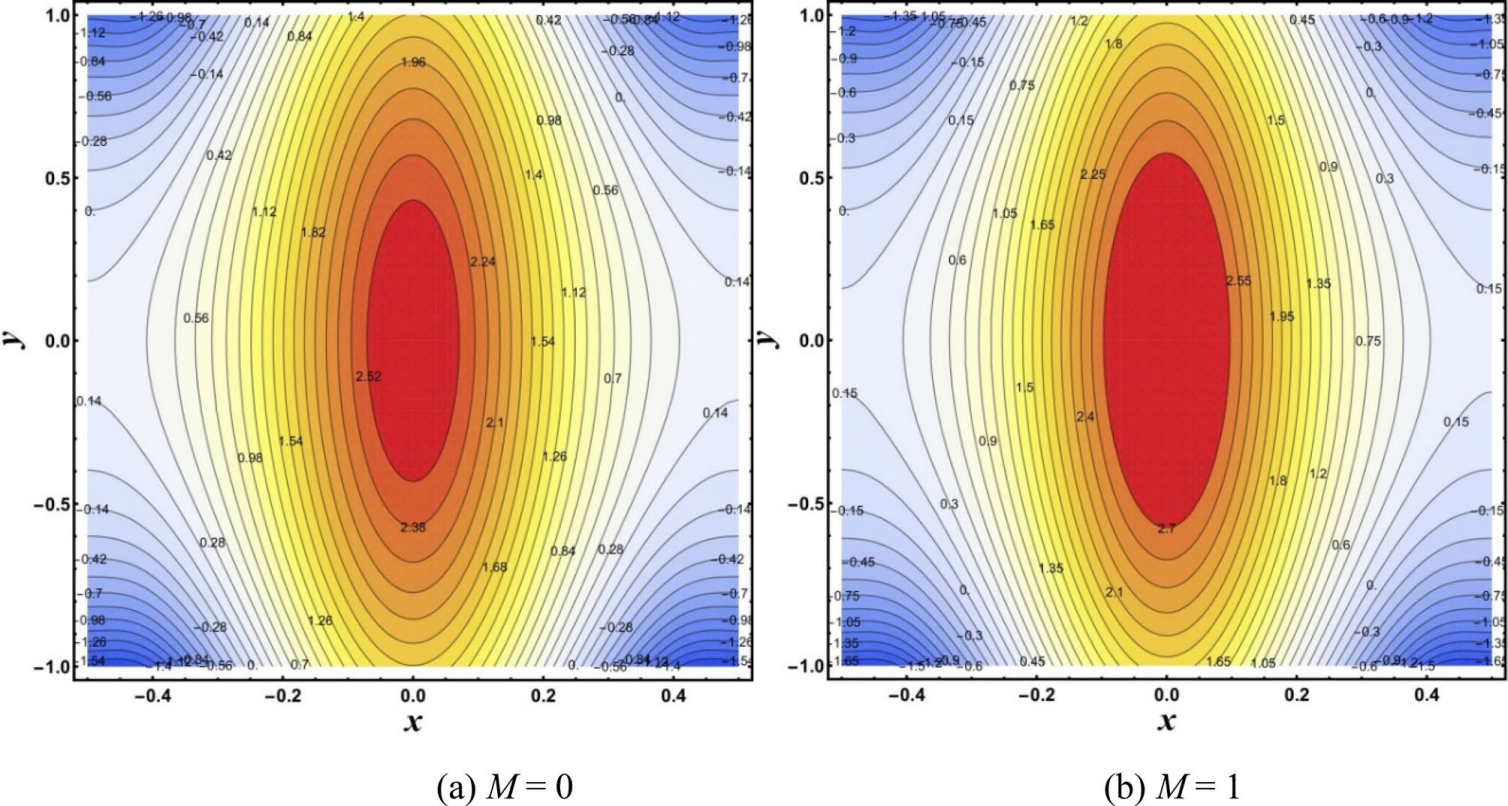
Isotherms for change in *M*.

**Figure 6:**
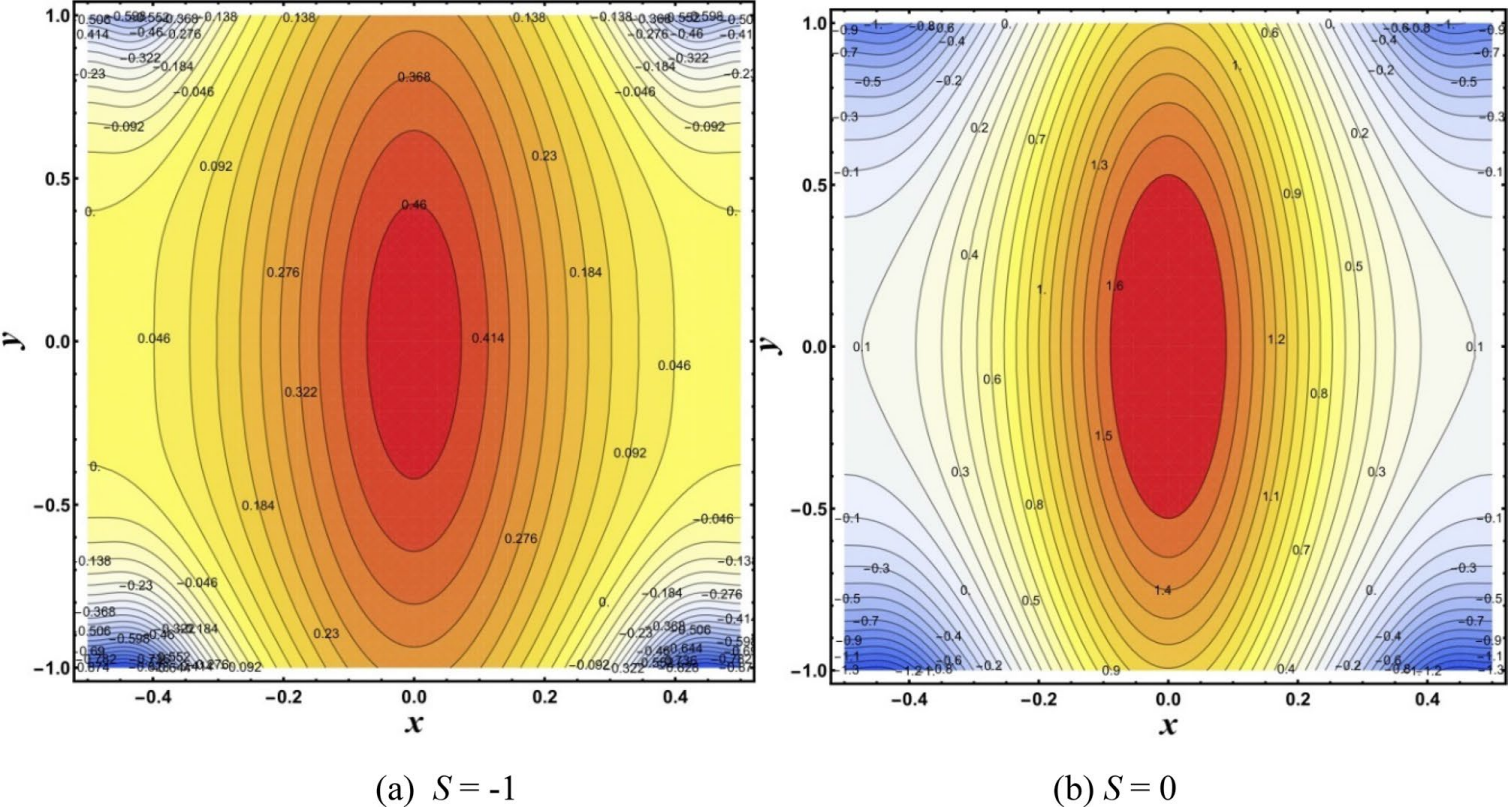
Isotherms for change in *S*.

The temperature distribution in the flow field is reflected by isotherms. Isotherms lines of modified hybrid nanofluid under the effects of *M* and *S* are drawn through Figs. 5,6. Figure 5a,b show that increasing *M* causes a notable change in the isotherms. It appears from Fig. 6A,B that the trapped bolus rises by increasing *S*.

### Velocity profile

**Figure 7:**
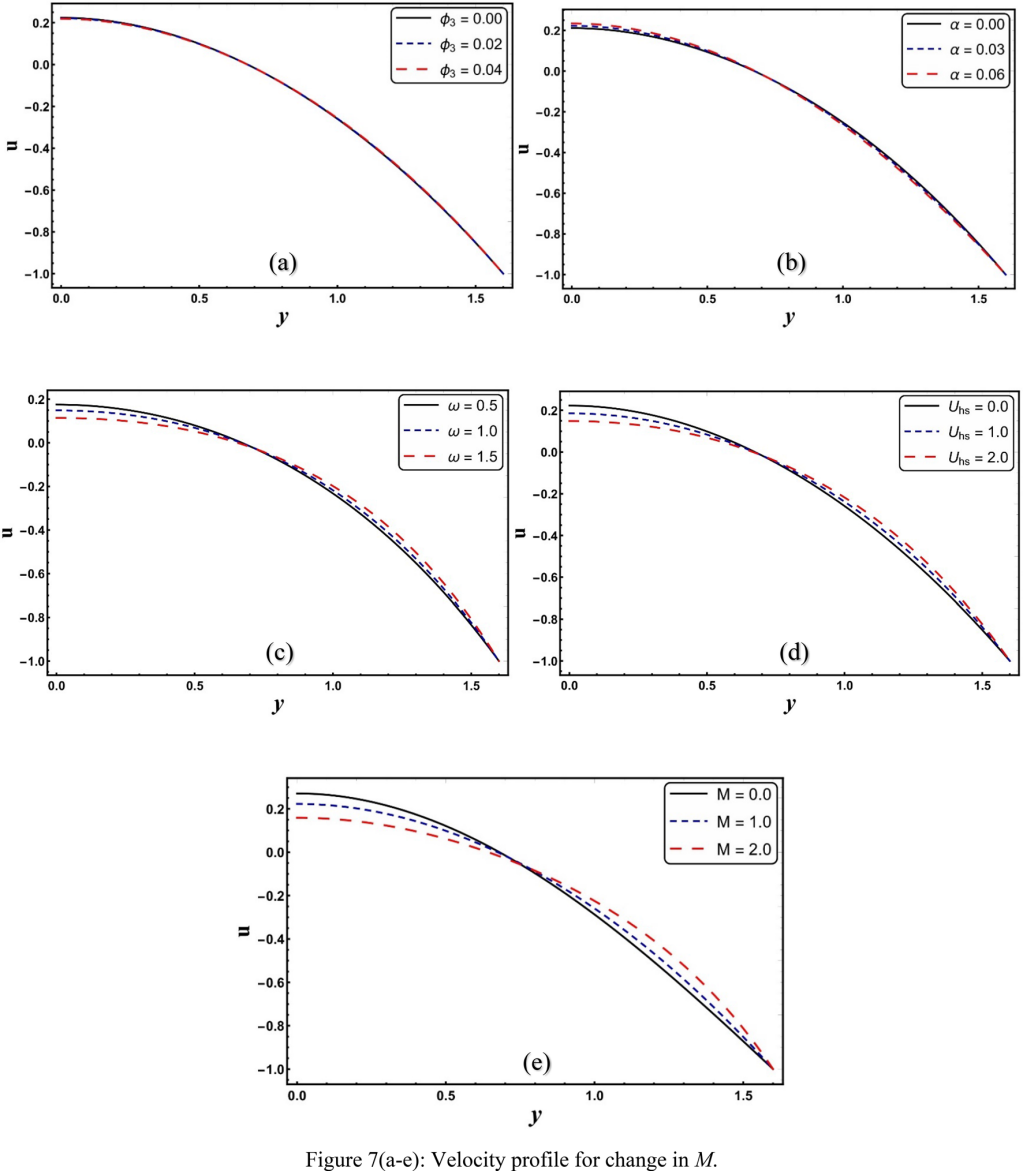
(**a**–**e**) Velocity profile for change in *M*.

Figure 7a–e are plotted to explore the response of velocity of (*Cu* + *CuO* + *Al*_2_*O*_3_) modified hybrid nanofluid against different involved parameters. Viewed from Fig. 7a that the magnitude of velocity of modified nanofluid reduces with increased NPs volume fraction. This is due to a higher volume fraction of aluminum oxide NPs (*ϕ*_*3*_), which increases the viscosity of the liquid, and therefore resists the movement of the liquid. This outcome is in accordance with Abbasi et al.^17^. Figure 7b illustrates that velocity grows with rising values of α. This means that the modified nanofluid, whose viscosity depends on temperature, reflects a higher velocity near the middle of the channel compared to the velocity of the nanofluid, which has a constant viscosity (α = 0). Figure 7c shows that increasing the electroosmotic parameter boosts nanofluid flow. The phenomenon of ELD affects an electroosmotic parameter. Velocity declines for larger *ω* when *U*_*hs*_ = −1.0. Figure 7d outlines that nanofluid flow is reduced by strengthening *U*_*hs*_. For auxiliary electric field velocity is higher and lower for the opposing electric field. *U*_*hs*_ is reliant on the electric field, which governs the flow in this case. The applied electric field has a direct relationship with *U*_*hs*_. Thus, at the positive value of *U*_*hs*_ it acts as a hindering force in the momentum equation, and at negative values it maintains the fluid flow. A similar behavior on velocity is also seen for higher Hartman number (see Fig. 7e). A Lorentz force is created in the flow when the Hartmann number (*M*) is increased, which causes the velocity to reduce.

### Pressure gradient

**Figure 8:**
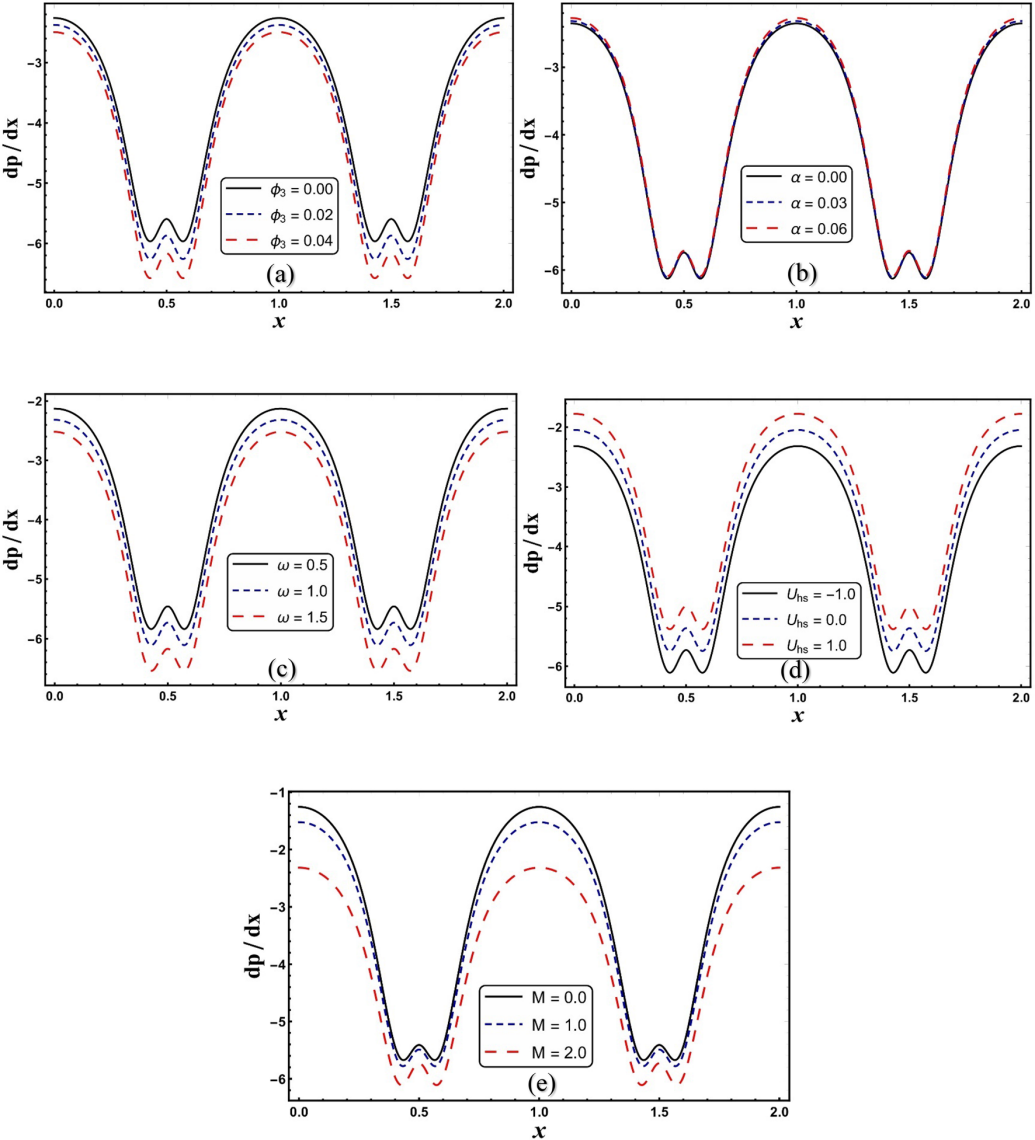
(**a**–**e**) Pressure gradient for change in *M.*

Figure 8a–e are made to evaluate the change in pressure gradient with respect to *x* across various embedded parameters. Figure 8a shows a reduction in pressure gradient with increasing concentration of nanomaterials. The addition of nanomaterials increases the resistance to fluid flow and thus reduces the pressure gradient. A reverse trend is seen in Fig. 8b with the impact of viscosity parameter. From Fig. 8c, pressure gradient decreases by improving electroosmotic parameter. The existence of EDL in charged surfaces abstains the flow, therefore pressure gradient decreases. Figure 8d portrays that pressure gradient develops by improving *U*_*hs*_. The pressure gradient is suppressed by increasing *M* (see Fig. 8e). The pressure gradient change to increase the Hartmann number is large when *M* > 1.

**Figure 9:**
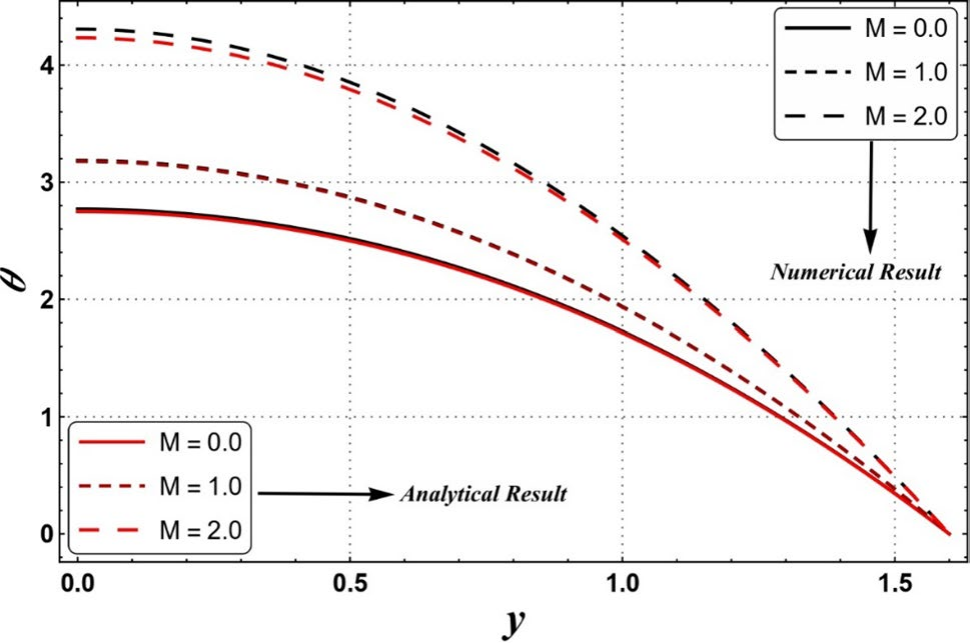
A comparison of the results obtained at analytical technique (HPM) and numerical technique (NDSolve).

A comparison between analytically (HPM) and numerical results (NDSolve) are also presented via Fig. 9. It is seen that both outcomes are consistent.

## Conclusions

The present endeavor brings out the collective effects of electro-magneto hydrodynamic, temperature dependent viscosity, homogeneous and heterogeneous chemical reaction rates in the peristaltic movement of a modified hybrid nanofluid containing *Al*_*2*_*O*_*3*_, *CuO*, *Cu* NPs in an aqueous solution. Significant observations are enumerated below:

- Heterogeneous reaction parameter aids for enhancing the concentration profiles while the homogeneous reaction parameter reduces the concentration.
- Temperature of modified HNFs significantly elevates with an increment in electroosmotic parameter.
- Rate of heat-transfer at the boundary is greater for blade-shaped NPs compared to other shaped NPs.
- Modified hybrid nanofluid has superior heat transfer rate relative to base fluid (*H*_*2*_*0*), ordinary nanofluid (*Al*_2_*O*_3_-*H*_*2*_*0*), hybrid nanofluid (*Cu* + *Al*_2_*O*_3_-*H*_*2*_*0*).
- The isotherms show a notable change when the Hartman number is increased.
- A development in pressure gradient is obtained by improving the Helmholtz-Smoluchowski velocity.

The results of this theoretical study can be extended by discussing it for various other Newtonian nanofluids through straight and curved channels. In addition, considering slippage conditions at the boundaries gives this study an accurate picture of reality.

## Data Availability

The datasets used and analyzed during the current study available from the corresponding author on reasonable request.

## Abbreviations

$$\overrightarrow{J}$$: Current density
${\phi }_{3}$: Volume fraction of *Al*_*2*_*O*_*3*_ nanoparticles
*β*_*f*_: Thermal expansion coefficient of fluid
*δ*: Wave number
${\phi }_{2}$: Volume fraction of *Cu* nanoparticles
*g*: Acceleration due to gravity
${\sigma }_{f}$: Electric conductivity of fluid
ε: Dimensionless heat generation/ absorption parameter
*T*_*w*_: Temperature at channel wall
*p*: Dimensionless pressure
*T*_0_: Temperature of wall
*F*: Dimensionless flow rate in wave frame
*Pr*: Prandtl number
*Re*: Reynolds number
*Br*: Brinkman number
*Ec*: Eckert number
*ρ*_*f*_: Density of fluid
*Gr*: Grashoff number
*M*: Hartman number
*θ*: Dimensionless temperature
*ψ*: Stream function
$\overrightarrow{B}$: Applied magnetic field
$\overrightarrow{E}$: Applied electric field
${\phi }_{1}$: Volume fraction of *Cuo* nanoparticles
*T*: Dimensional temperature
Φ: Dimensional heat generation/absorption parameter
*η*: Dimensionless flow rate in laboratory frame
